# Safety and usage of darbepoetin alfa in children with chronic kidney disease: prospective registry study

**DOI:** 10.1007/s00467-015-3225-0

**Published:** 2015-10-19

**Authors:** Franz Schaefer, Bernd Hoppe, Therese Jungraithmayr, Günter Klaus, Lars Pape, Mourad Farouk, Janet Addison, Nick Manamley, Karel Vondrak

**Affiliations:** University of Heidelberg, Heidelberg, Germany; University Hospital Bonn, Bonn, Germany; Medical University of Innsbruck, Innsbruck, Austria; University of Marburg, Marburg, Germany; Hannover Medical School, Hannover, Germany; Amgen (Europe) GmbH, Zug, Switzerland; Amgen Ltd, Cambridge, UK; University Hospital Motol, Prague, Czech Republic; Division of Pediatric Nephrology, Center for Pediatrics and Adolescent Medicine, Im Neuenheimer Feld 430, 69120 Heidelberg, Germany

**Keywords:** Adverse events, Anemia, Chronic kidney disease, Darbepoetin alfa, Hemoglobin, Pediatric

## Abstract

**Background:**

Limited prospective data are available on the long-term safety of darbepoetin alfa (DA) for treating anemia in children with chronic kidney disease (CKD).

**Methods:**

In this prospective, phase IV, observational registry study, children ≤16 years of age with CKD anemia and receiving DA were observed for ≤2 years. Adverse events (AEs), DA dosing, hemoglobin (Hb) concentrations, and transfusions were recorded.

**Results:**

A total of 319 patients were included in the analysis (mean age, 9.1 years), 158 (49.5 %) of whom were on dialysis at study entry. Of 434 serious AEs reported in 162 children, the most common were peritonitis (10.0 %), gastroenteritis (6.0 %), and hypertension (4.1 %). Six patients (1.9 %) died (unrelated to DA). Four patients (1.3 %) experienced six serious adverse drug reactions. The geometric mean DA dose range was 1.4–2.0 μg/kg/month. Mean baseline Hb concentration was 11.1 g/dl; mean values for children receiving and not receiving dialysis at baseline ranged between 10.9 and 11.5 g/dl and 11.2–11.7 g/dl, respectively. Overall, 48 patients (15.0 %) received ≥1 transfusion.

**Conclusions:**

No new safety signals for DA were identified in children receiving DA for CKD anemia for ≤2 years. Based on Hb concentrations and transfusion requirements, DA was effective at managing anemia in these patients.

## Introduction

Although the prevalence of chronic kidney disease (CKD) in children is much lower than in adults, the condition is associated with substantial mortality and morbidity in the pediatric population. Despite evidence of improved long-term survival rates, mortality in children and adolescents is 55 times greater in those on renal replacement therapy than in children without end-stage renal disease [1, 2]. Hypertension and anemia are common complications [3–7]. In a large cohort of North American children aged ≥2 years with CKD and not on dialysis, anemia prevalence increased from 18.5 % in those at stage II to 68 % of those at stage V, and the presence of anemia significantly increased the risk of hospitalization [8].

Anemia in children with CKD can be corrected with iron supplements and erythropoiesis-stimulating agents (ESAs), although some evidence suggests these treatments may be under-utilized. In CKD patients receiving dialysis, for example, relatively fewer children than adults achieve target hemoglobin (Hb) [9], and in 1,724 children and adolescents in the North American Pediatric Renal Transplant Cooperative Study database, of whom 31 % had anemia (hematocrit <30 %), only 13 % were receiving erythropoietin [4].

Currently, darbepoetin alfa (DA) is indicated for the treatment of anemia in adults and children with CKD, whether or not they are receiving dialysis [10]. DA is administered either subcutaneously (s.c.) or intravenously (i.v.) with the aim of increasing Hb to a value between 10 and 12 g/dl, or between 9.5 and 11.5 g/dl if < 2 years of age [11]. Dosing recommendations for children aged ≥11 years are very similar to those for adults, but recommendations for children aged <11 years are not yet available. In an open-label study of children aged 1–18 years with an estimated glomerular filtration rate (eGFR) of <30 ml/min × 1.73 m^2^, DA was found to be non-inferior to recombinant human erythropoietin (rHuEpo) for treating anemia [12], but the follow-up in this and other studies that evaluated the use of DA in children aged <11 years [13–16] was limited to 28 weeks.

Here, we present the final results of a prospective observational registry study that aimed to evaluate the safety of DA in anemic children with CKD over a longer period (2 years), and to investigate dosage patterns and Hb concentrations over time.

## Methods

### Study design and patient selection

This prospective phase IV observational registry study (NCT00838097) enrolled patients from 37 pediatric nephrology centers in 13 European countries from February 2008 to February 2011. The centers all had experience in treating pediatric CKD patients with DA in routine clinical practice.

To be eligible for inclusion, children and adolescents aged 0–16 years with anemia attributed to CKD had to either be receiving dialysis or have an eGFR of <60 ml/min × 1.73 m^2^ for at least 3 months, and be receiving DA at the time of study enrollment. Patients were excluded if they had active malignancy or were receiving chemotherapy or radiotherapy.

Patients were followed up for a maximum of 2 years, or until the time of study withdrawal if this occurred earlier. Protocol-specified reasons for premature study withdrawal included permanent cessation of DA treatment, renal transplantation, enrollment into an interventional study, or withdrawal of informed consent. Reasons for withdrawal and for stopping DA treatment were recorded.

In accordance with the observational design, the DA treatment regimen was not pre-specified and treating physicians could alter treatment dosage or temporarily suspend treatment at any time.

### Data collection

Baseline data collected for each patient, if available, included demographic and clinical characteristics, medical history (including history and cause of CKD and time of diagnosis, transplant and dialysis history, co-morbidities, red blood cell [RBC] transfusions in the previous year and history of ESA use), dosing information for DA (date of first use, dose, frequency, and administration route), and Hb concentration levels at entry and during the 3 previous months. At 3-monthly intervals thereafter, the key parameters recorded were DA dosing information, Hb concentrations, information on dialysis if started since the previous assessment, renal transplants and RBC transfusions received, details of iron supplementation, and hospitalizations. All data available from each patient’s medical record were abstracted to electronic case report forms (eCRFs).

All adverse events (AEs) were recorded in the eCRF from the time of informed consent to the end of the study period. The three co-primary endpoints were the incidences of serious AEs (SAEs), serious adverse drug reactions (SADRs; i.e., SAEs considered by the treating physician to be associated with exposure to DA), and the following protocol-specified events of medical interest (EMIs): thromboembolic events, seizures, severe hypertension, cardiovascular events (e.g., arrhythmia and heart failure), pure red cell aplasia, and hypersensitivity reactions. For severe hypertension, no threshold blood pressure (BP) value was pre-defined; these events were reported at the discretion of the treating physician. The relationship of any AE to DA treatment was also assessed by the treating physician. Secondary endpoints were the incidence of non-SADRs, and the DA dose and Hb concentrations over time.

### Statistical analyses

No formal hypotheses were tested. Patient disposition, demographic and clinical characteristics, medical history, and previous ESA therapy were summarized descriptively. For the analysis of SAEs, SADRs, and EMIs, exposure-adjusted incidence rates and incidence rates based on life-table methodology were summarized together with 95 % confidence intervals (CIs), and the number of patients affected were summarized by preferred term and system organ class. The population used for all analyses was the full analysis set (FAS), i.e., all patients who received at least one dose of DA. Analyses were also done for four age subgroups (<1 year, 1–5 years, 6–11 years, and ≥12 years) and two dialysis subgroups (on dialysis at baseline or not). Non-SADRs were summarized by preferred term and system organ class.

The actual and weight-adjusted doses of DA were summarized monthly using descriptive statistics, as well as the cumulative DA dose over the duration of the study. Changes in DA dose (actual and weight-adjusted) from baseline were also summarized descriptively. Hb concentration was summarized at baseline and at 3-monthly periods. RBC transfusions were summarized by tabulating the incidence of transfusions as well as the number of transfusions received per patient over the study duration. Information on body weight, BP, and other laboratory test data were also collected (data not shown).

## Results

### Patients and treatment

**Fig. 1:**
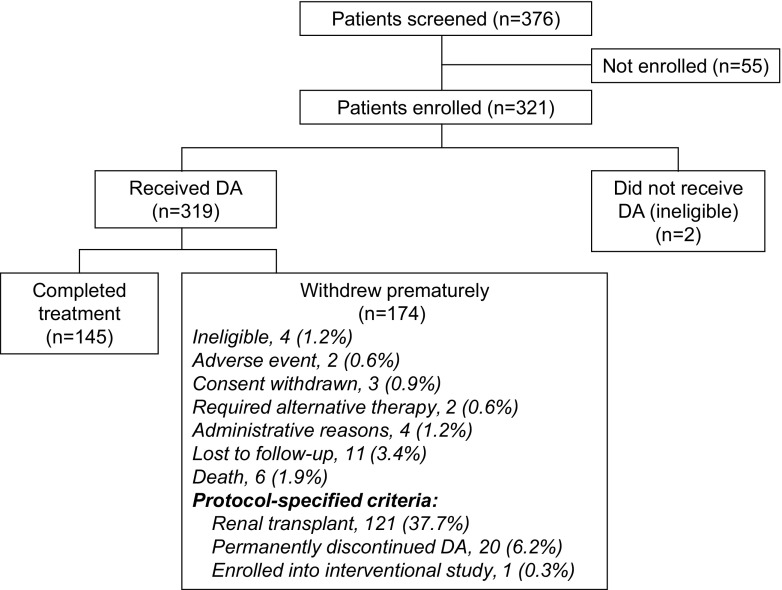
Patient disposition. Reasons for premature withdrawal are shown as % of all patients enrolled. *DA* darbepoetin alfa

Of the 321 patients who entered the study, 145 (45.2 %) patients completed the study, and 176 (54.8 %) withdrew early, most commonly because of renal transplantation (121 [37.7 %] patients). Twenty patients (6.2 %) stopped DA treatment permanently, 11 (3.4 %) were lost to follow-up, and six patients (1.9 %) died. The reasons for study withdrawal in the other 16 patients are shown in Fig. 1. Two of the 321 patients who entered did not receive DA; the remaining 319 were included in the FAS. The median duration of follow-up (from first dose to the end of the study) was 88 weeks (range, 1–117 weeks); the median duration of treatment (from the first to the last dose of DA) was the same. A marked difference was seen in follow-up duration between those on dialysis at baseline and those who were not (medians of 57 and 102 weeks, respectively).

The mean age of patients in the FAS was 9.1 (standard deviation [SD], 5.0) years: the largest age subgroup was children aged ≥12 years (*n* = 133) and the smallest was infants aged <1 year (*n* = 13) (Table 1). The majority of patients were male (55.5 %). Of the specified primary causes of CKD, the most common were glomerulopathies (24.8 %), renal hypoplasia/dysplasia (23.2 %), obstructive nephropathy (11.6 %), and polycystic kidney disease (7.8 %) (Table 1). The distribution of etiologies varied very little by age subgroup.

**Table 1:** Baseline demographics and clinical characteristics in the full analysis set

	All patients (*n* = 319)	Age <1 year (*n* = 13)	Age 1–5 years (*n* = 83)	Age 6–11 years (*n* = 90)	Age ≥12 years (*n* = 133)
Male patients, *n* (%)	177 (55.5)	8 (61.5)	49 (59.0)	49 (54.4)	71 (53.4)
Age at diagnosis of CKD, years (mean and SD; *n* = 317)	3.6 (4.5)	0.1 (0.2)	1.0 (1.4)	3.5 (3.4)	5.8 (5.4)
Race, *n* (%)					
White/Caucasian	274 (85.9)	13 (100)	75 (90.4)	77 (85.6)	109 (82.0)
Black/African American	9 (2.8)	0	0	3 (3.3)	6 (4.5)
Asian	8 (2.5)	0	3 (3.6)	4 (4.4)	1 (0.8)
Other	9 (2.8)	0	3 (3.6)	2 (2.2)	4 (3.0)
Not recorded	19 (6.0)	0	2 (2.4)	4 (4.4)	13 (9.8)
Primary etiology of CKD, *n* (%)					
Glomerulopathies	79 (24.8)	1 (7.7)	20 (24.1)	22 (24.4)	36 (27.1)
Renal dysplasia/hypoplasia	74 (23.2)	2 (15.4)	23 (27.7)	24 (26.7)	25 (18.8)
Obstructive nephropathy	37 (11.6)	3 (23.1)	10 (12.0)	5 (5.6)	19 (14.3)
Polycystic kidney disease	25 (7.8)	2 (15.4)	12 (14.5)	7 (7.8)	4 (3.0)
Reflux nephropathy	20 (6.3)	2 (15.4)	2 (2.4)	7 (7.8)	9 (6.8)
Nephronophthisis	18 (5.6)	1 (7.7)	1 (1.2)	2 (2.2)	14 (10.5)
Hemolytic uremic syndrome	17 (5.3)	0	3 (3.6)	10 (11.1)	4 (3.0)
Post-ischemic nephropathy	11 (3.5)	1 (7.7)	5 (6.0)	4 (4.4)	1 (0.8)
Other specified causes	24 (7.5)	1 (7.7)	5 (6.0)	6 (6.7)	12 (9.0)
Unknown or not recorded	14 (4.4)	0	2 (2.4)	3 (3.3)	9 (6.8)
Baseline hemoglobin concentration, *n* (%)					
< 10 g/dl	70 (21.9)	3 (23.1)	18 (21.7)	20 (22.2)	29 (21.8)
10–12 g/dl	161 (50.5)	6 (46.2)	41 (49.4)	50 (55.6)	64 (48.1)
> 12 g/dl	77 (24.1)	3 (23.1)	21 (25.3)	18 (20.0)	35 (26.3)
Missing	11 (3.4)	1 (7.7)	3 (3.6)	2 (2.2)	5 (3.8)
ESA use at study entry, *n* (%)					
Intravenous DA	80 (25.1)	2 (15.4)	11 (13.3)	20 (22.2)	47 (35.3)
Subcutaneous DA	219 (68.7)	10 (76.9)	63 (75.9)	64 (71.1)	82 (61.7)
Not on DA^a^	20 (6.2)	1 (7.7)	9 (10.8)	6 (6.7)	4 (3.0)
Previous kidney transplants, *n* (%)					
≥1 RBC transfusion in previous year, *n* (%)	66 (20.7)	0	7 (8.4)	19 (21.1)	40 (30.1)
Dialysis status at baseline	57 (17.9)	3 (23.1)	19 (22.9)	15 (16.7)	20 (15.0)
Conservative treatment (no dialysis)	160 (50.2)	6 (46.2)	42 (50.6)	48 (53.3)	64 (48.1)
Hemodialysis	74 (23.2)	1 (7.7)	11 (13.3)	18 (20.0)	44 (33.1)
Peritoneal dialysis	84 (26.3)	6 (46.2)	30 (36.1)	24 (26.7)	24 (18.0)
Unknown	1 (0.3)	0	0	0	1 (0.8)

*CKD* chronic kidney disease; *DA* darbepoetin alfa; *ESA* erythropoiesis stimulating agent; *RBC* red blood cells; *SD* standard deviation

^a^Protocol violation

Hypertension was the most common co-morbidity in the FAS, experienced currently or previously by 155 (48.6 %) patients, 137 of whom were receiving antihypertensive treatment at baseline. Other co-morbidities were less common, mostly affecting <10 % of patients (except for neurological conditions: 55 [17.2 %] patients).

At baseline, the mean Hb concentration for all patients with available data (*n* = 308) was 11.1 (SD, 1.6) g/dl: the mean value was lower in children aged <1 year (10.6 g/dl) than in the other three age subgroups (11.0–11.2 g/dl). Baseline Hb values were within the 10–12 g/dl range in 161 (50.5 %) of the patients; the number of patients with baseline Hb values above 12 g/dl (77; 24.1 %) or below 10 g/dl (70; 21.9 %) were similar. Of 319 patients in the FAS, 158 (49.5 %) were on dialysis at study entry. The proportions on hemodialysis and peritoneal dialysis in the FAS were similar (74 [23.2 %] and 84 [26.3 %], respectively), but varied by age subgroup, with older children generally more likely to be on hemodialysis than younger children (Table 1). In 158 patients with available data, the median duration of dialysis before study entry was 9.7 months (range, 0–190 months). Sixty-six (20.7 %) patients had previously received a kidney transplant. The mean (SD) eGFR at baseline in the 142 non-dialysis patients was 37 (23) ml/min × 1.73 m^2^.

During the 12 months before study entry, 267 patients (83.7 %) had been treated with DA, 98 (30.7 %) had received epoetin-beta, 13 (4.1 %) had received epoetin-alfa, and 16 (5.0 %) were ESA-naive. At study entry, 299 patients (93.7 %) were being treated with DA, with a geometric mean dose of 52.6 μg/month, and a weight-adjusted value of 1.99 μg/kg/month (95 % CI: 1.82–2.18; *n* = 273). The weight-adjusted geometric mean doses at baseline for i.v. and s.c. routes, respectively, were 2.89 μg/kg/month (95 % CI: 2.43–3.43) and 1.75 μg/kg/month (95 % CI: 1.58–1.93). The 20 patients who were not receiving DA at enrollment started treatment 2–84 days (median, 9 days) after enrollment; although classed as protocol violators, they were included in all analyses.

### Tolerability and safety

#### Serious adverse events and deaths

A total of 162 (50.8 %) patients reported a total of 434 SAEs, equating to an exposure-adjusted event rate (EAER) of 101.8 per 100 person-years [95 % CI: 92.5–111.9]. The three most common SAEs were peritonitis (32 patients; 10.0 % of FAS), gastroenteritis (19 patients; 6.0 %), and hypertension (13 patients; 4.1 %). All SAEs of peritonitis (44 events) were reported by patients who were on dialysis (mostly peritoneal) at the time of the event. Details are shown in Tables 2, 3, and 4.

**Table 2:** Most common serious adverse events in the full analysis set (those with at least five events)

	All patients (*n* = 319)		
	Patients reporting, *n* (%)	Events, *n*	EAER/100 person-years (95 % CI)
Any SAE	162 (50.8)	434	101.8 (92.5–111.9)
Peritonitis	32 (10.0)	44	10.3 (7.5–13.9)
Hypertension	13 (4.1)	23	5.4 (3.4–8.1)
Gastroenteritis	19 (6.0)	22	5.2 (3.2–7.8)
Convulsions	10 (3.1)	16	3.8 (2.2–6.1)
Diarrhea	8 (2.5)	11	2.6 (1.3–4.6)
Vomiting	8 (2.5)	8	1.9 (0.8–3.7)
Urinary tract infection	7 (2.2)	8	1.9 (0.8–3.7)
Renal failure chronic	7 (2.2)	7	1.6 (0.7–3.4)
Sepsis	7 (2.2)	7	1.6 (0.7–3.4)
Pneumonia	6 (1.9)	7	1.6 (0.7–3.4)
Pyelonephritis	6 (1.9)	7	1.6 (0.7–3.4)
Pyrexia	4 (1.3)	7	1.6 (0.7–3.4)
Medical device complication	4 (1.3)	6	1.4 (0.5–3.1)
Bronchitis	6 (1.9)	6	1.4 (0.5–3.1)
Catheter site infection	6 (1.9)	6	1.4 (0.5–3.1)
Device-related infection	6 (1.9)	6	1.4 (0.5–3.1)
Dehydration	6 (1.9)	6	1.4 (0.5–3.1)
Hypertensive crisis	5 (1.6)	6	1.4 (0.5–3.1)
Abdominal pain	3 (0.9)	5	1.2 (0.4–2.7)
Upper respiratory tract infection	4 (1.3)	5	1.2 (0.4–2.7)
Arteriovenous fistula thrombosis	5 (1.6)	5	1.2 (0.4–2.7)
Hyperkalemia	3 (0.9)	5	1.2 (0.4–2.7)

*CI* confidence interval; *EAER* exposure-adjusted event rate; *SAE* serious adverse event

**Table 3:** Most common serious adverse events in the full analysis set (those with exposure-adjusted event rates per 100 person-years of >1) by age subgroup

	Infants aged <1 year (*n* = 13)		Children aged 1–5 years (*n* = 83)		Children aged 6–11 years (*n* = 90)		Children aged ≥12 years (*n* = 133)	
	Patients reporting, *n* (%)	EAER/100 person-years (95 % CI)	Patients reporting, *n* (%)	EAER/100 person-years (95 % CI)	Patients reporting, *n* (%)	EAER/100 person-years (95 % CI)	Patients reporting, *n* (%)	EAER/100 person-years (95 % CI)
Any SAE	7 (53.8)	149.0 (94.4–223.5)	51 (61.4)	152.8 (130.3–178.1)	39 (43.3)	69.0 (55.4–85.0)	65 (48.9)	90.4 (76.9–105.6)
Peritonitis	1 (7.7)	13.0 (1.6–46.8)	13 (15.7)	16.8 (9.9–26.5)	10 (11.1)	11.0 (6.0–18.4)	8 (6.0)	5.7 (2.7–10.5)
Hypertension	1 (7.7)	6.5 (0.16–36.1)	6 (7.2)	10.3 (5.1–18.3)	4 (4.4)	3.1 (0.9–8.0)	2 (1.5)	4.0 (1.6–8.2)
Gastroenteritis	2 (15.4)	13.0 (1.6–46.8)	8 (9.6)	8.4 (3.8–15.9)	2 (2.2)	1.6 (0.2–5.7)	7 (5.3)	5.1 (2.3–9.7)
Convulsions	1 (7.7)	25.9 (7.1–66.3)	4 (5.8)	4.7 (1.5–10.9)	4 (4.4)	4.7 (1.7–10.2)	1 (0.8)	0.6 (0.0–3.2)
Diarrhea	1 (7.7)	6.5 (0.16–36.1)	1 (1.2)	1.9 (0.2–6.7)	2 (2.2)	1.6 (0.2–5.7)	4 (3.0)	3.4 (1.3–7.4)
Vomiting	0	0	4 (5.8)	3.7 (1.0–9.5)	1 (1.1)	0.8 (0.0–4.4)	3 (2.3)	1.7 (0.4–5.0)
Urinary tract infection	0	0	3 (3.6)	2.8 (0.6–8.2)	0	0	4 (3.0)	2.8 (0.9–6.6)
Renal failure chronic	0	0	5 (6.0)	4.7 (1.5–10.9)	0	0	2 (1.5)	1.1 (0.1–4.1)
Sepsis	0	0	3 (3.6)	2.8 (0.6–8.2)	1 (1.1)	0.8 (0.0–4.4)	3 (2.3)	1.7 (0.4–5.0)
Pneumonia	0	0	1 (1.2)	0.9 (0.0–5.2)	2 (2.2)	2.4 (0.5–6.9)	3 (2.3)	1.7 (0.4–5.0)
Pyelonephritis	0	0	0	0	1 (1.1)	0.8 (0.0–4.4)	5 (3.8)	3.4 (1.3–7.4)
Pyrexia	1 (7.7)	6.5 (0.16–36.1)	3 (3.6)	5.6 (2.1–12.2)	0	0	0	0
Medical device complication	0	0	2 (2.4)	3.7 (1.0–9.5)	1 (1.1)	0.8 (0.0–4.4)	1 (0.8)	0.6 (0.0–3.2)
Bronchitis	0	0	3 (3.6)	2.8 (0.6–8.2)	3 (3.3)	2.4 (0.5–6.9)	0	0
Catheter site infection	2 (15.4)	13.0 (1.6–46.8)	1 (1.2)	0.9 (0.0–5.2)	1 (1.1)	0.8 (0.0–4.4)	2 (1.5)	1.1 (0.1–4.1)
Device-related infection	1 (7.7)	6.5 (0.16–36.1)	2 (2.4)	1.9 (0.2–6.7)	1 (1.1)	0.8 (0.0–4.4)	2 (1.5)	1.1 (0.1–4.1)
Dehydration	0	0	5 (6.0)	4.7 (1.5–10.9)	0	0	1 (0.8)	0.6 (0.0–3.2)
Hypertensive crisis	0	0	0	0	0	0	5 (3.8)	3.4 (1.3–7.4)
Abdominal pain	0	0	1 (1.2)	2.8 (0.6–8.2)	2 (2.2)	1.6 (0.2–5.7)	0	0
Upper respiratory tract infection	0	0	3 (3.6)	3.7 (1.0–9.5)	0	0	1 (0.8)	0.6 (0.0–3.2)
Arteriovenous fistula thrombosis	0	0	0	0	1 (1.1)	0.8 (0.0–4.4)	4 (3.0)	2.3 (0.6–5.8)
Hyperkalemia	0	0	2 (2.4)	3.7 (1.0–9.5)	1 (1.1)	0.8 (0.0–4.4)	0	0

*CI* confidence interval; *EAER* exposure-adjusted event rate; *SAE* serious adverse event

**Table 4:** Most common serious adverse events in the full analysis set (those with exposure-adjusted event rates per 100 person-years of >1) broken down for subgroups receiving and not receiving dialysis at baseline (data for one patient with unknown dialysis status at baseline not shown)

	Children not on dialysis (*n* = 160)		Children on dialysis (*n* = 158)	
	Patients reporting, *n* (%)	EAER/100 person-years (95 % CI)	Patients reporting, *n* (%)	EAER/100 person-years (95 % CI)
Any SAE	77 (48.1)	73.8 (63.5–85.3)	85 (53.8)	140.3 (123.5–158.8)
Peritonitis	7 (4.4)	3.2 (1.4–6.4)	25 (15.8)	20.1 (14.0–27.8)
Hypertension	2 (1.3)	1.6 (0.4–4.2)	11 (7.0)	10.6 (6.4–16.5)
Gastroenteritis	11 (6.9)	5.7 (3.1–9.5)	8 (5.1)	4.5 (1.9–8.8)
Convulsions	4 (2.5)	1.6 (0.4–4.2)	6 (3.8)	6.7 (3.5–11.7)
Diarrhea	3 (1.9)	2.0 (0.7–4.7)	5 (3.2)	3.3 (1.2–7.3)
Vomiting	1 (0.6)	0.4 (0.0–2.3)	7 (4.4)	3.9 (1.6–8.0)
Urinary tract infection	6 (3.8)	2.8 (1.1–5.9)	1 (0.6)	0.6 (0.0–3.1)
Renal failure chronic	6 (3.8)	2.4 (0.9–5.3)	1 (0.6)	0.6 (0.0–3.1)
Sepsis	4 (2.5)	1.6 (0.4–4.2)	3 (1.9)	1.7 (0.3–4.9)
Pneumonia	4 (2.5)	1.6 (0.4–4.2)	2 (1.3)	1.7 (0.3–4.9)
Pyelonephritis	4 (2.5)	1.6 (0.4–4.2)	2 (1.3)	1.7 (0.3–4.9)
Pyrexia	1 (0.6)	0.4 (0.0–2.3)	3 (1.9)	3.3 (1.2–7.3)
Medical device complication	0	0	4 (2.5)	3.3 (1.2–7.3)
Bronchitis	4 (2.5)	1.6 (0.4–4.2)	2 (1.3)	1.1 (0.1–4.0)
Catheter site infection	0	0	6 (3.8)	3.3 (1.2–7.3)
Device-related infection	0	0	6 (3.8)	3.3 (1.2–7.3)
Dehydration	4 (2.5)	1.6 (0.4–4.2)	2 (1.3)	1.1 (0.1–4.0)
Hypertensive crisis	2 (1.3)	0.8 (0.1–2.9)	3 (1.9)	2.2 (0.6–5.7)
Abdominal pain	2 (1.3)	0.8 (0.1–2.9)	1 (0.6)	1.7 (0.3–4.9)
Upper respiratory tract infection	0	0	4 (2.5)	2.8 (0.9–6.5)
Arteriovenous fistula thrombosis	1 (0.6)	0.4 (0.0–2.3)	4 (2.5)	2.2 (0.6–5.7)
Hyperkalemia	1 (0.6)	0.8 (0.1–2.9)	2 (1.3)	1.7 (0.3–4.9)

*CI* confidence interval; *EAER* exposure-adjusted event rate; *SAE* serious adverse event

Six patients (1.9 %) had fatal AEs during the study. The fatal AEs in the younger children were gastrointestinal necrosis, pulmonary edema, and severe hypertension leading to cardiac arrest (in patients aged 15, 11, and 6 months, respectively), and in the three older children, the causes of death were congenital mitochondrial cytopathy, pulmonary edema, and sepsis (in patients aged 5, 15, and 7 years old, respectively). No fatal AE was considered to be related to DA administration.

#### Adverse drug reactions

Four patients (1.3 %) experienced six SADRs: arteriovenous fistula thrombosis and embolism (single episode), priapism, thrombocytopenia, hemolysis, hemolytic anemia, and partial blindness. Two patients were in the 6–11 years age group, and two in the ≥12 years group (Table 5). Injection site pain was reported as a non-SADR by four patients (1.3 %), aged 1, 6, 10, and 12 years old.

**Table 5:** Details of serious adverse drug reactions

Patient number	Age (years)	Event(s)	Time of event (day of treatment)	Outcome	EAER (95 % CI)	Hemoglobin concentration	DA dose
164004	10	Arteriovenous fistula thrombosis and embolism (single episode)^a^	Day 46	Thrombosed fistula closed, new fistula created	0.8 (0.0–0.4) for each	10.6 g/dl (60 days before events)	10 μg QW; withheld on days 47–71
143011	11	Priapism (3 episodes)	Days 468, 482, and 489	Each episode resolved within 1 day	2.4 (0.5–6.9)	8.3 g/dl (day 468) 7.6 g/dl (day 473)	30 μg Q2W, days 1–482; reduced to 30 μg QW on day 483
103015	14	Thrombocytopenia Hemolysis Hemolytic anemia	Day 22 Day 22 Day 22	Resolved 85 days after diagnosis Resolved 175 days after diagnosis Resolved 175 days after diagnosis	0.6 (0.0–3.2) 0.6 (0.0–3.2) 0.6 (0.0–3.2)	9.4 g/dl	30 μg QW; withheld from ∼ day 29 to day 107
103008	15	Partial blindness	Day 3	Resolved ∼2 weeks after DA discontinued	0.6 (0.0–3.2)	6.3, 6.6, & 6.6 g/dl on days 1–3	20 μg twice weekly; discontinued on day 3

*DA* darbepoetin alfa; *EAER* exposure-adjusted event rate per 100 person-years; *QW* once weekly; *Q2W* every 2 weeks

^a^This event was originally reported as a single episode, although described as two separate terms in the Clinical Study Report

#### Events of medical interest

A total of 39 patients (12.2 %) reported a total of 82 EMIs in four categories: severe hypertension (34 events in 21 patients), seizures (24 in 14 patients), thromboembolic/cardiovascular events (21 in 12 patients), and hypersensitivity reactions (three in three patients). The EAER per 100 person-years for all EMIs was 19.2 [95 % CI: 15.3–23.9]. The three most commonly reported EMIs were hypertension (15 patients; 4.7 %), convulsions (ten patients; 3.1 %), and hypertensive crisis (five patients; 1.6 %). The EAERs for these three events were 5.9 (95 % CI: 3.8–8.7), 3.8 (2.2–6.1), and 1.4 (0.5–3.1), respectively. One patient had simultaneous convulsions and hypertensive crisis. All other EMIs were reported by either one or two patients (EAERs, 0.2 [0.0–1.3] and 0.5 [0.1–1.7]). The proportion of children reporting EMIs was higher in the three younger age subgroups (14–15 %) than the oldest group (9 %).

### Darbepoetin alfa dose

**Fig. 2:**
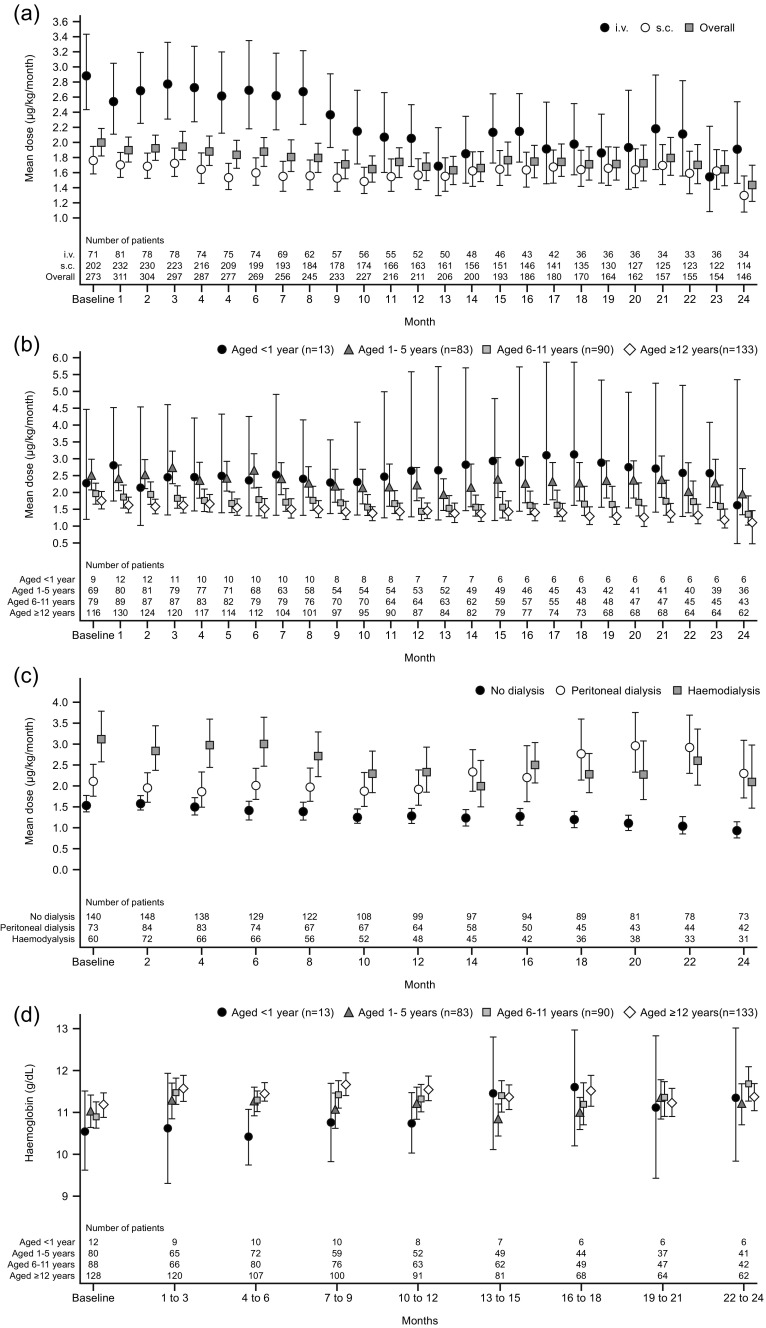
**a** Weight-adjusted geometric mean dose of darbepoetin alfa per month by administration route (full analysis set); *error bars* show 95 % confidence intervals. i.v., intravenous; s.c., subcutaneous. **b** Weight-adjusted geometric mean dose of darbepoetin alfa per month by age subgroup (full analysis set); *error bars* show 95 % confidence intervals. **c** Weight-adjusted geometric mean dose of darbepoetin alfa per month by dialysis status at two-monthly intervals (full analysis set). **d** Mean hemoglobin concentrations for each age subgroup (full analysis set); *error bars* show 95 % confidence intervals

The overall geometric mean monthly weight-adjusted DA dose was relatively stable over time, ranging from 1.6 to 2.0 μg/kg from baseline to month 12 (*n* = 211–273) and from 1.4 to 1.8 μg/kg in the second 12 months (*n* = 146–206). The mean i.v. dose range was higher than the s.c. dose range, particularly in the first 9 months (Fig. 2a). Over the full study period, children on dialysis at baseline received higher geometric mean DA doses than those who were not (1.9–2.7 μg/kg and 1.3–1.6 μg/kg, respectively) and were more likely to receive i.v. dosing than those not on dialysis: 58/124 (46.7 %) and 16/145 (11.0 %), respectively, at month 6 and 37/90 (41.1 %) and 15/121 (12.4 %), respectively, at month 12.

Over the course of the study, the younger children received higher geometric mean weight-adjusted DA doses (1.7–3.2 μg/kg/month for those aged <1 year; 1.9–2.7 μg/kg/month for those aged 1–5 years) than the older children (1.5–2.0 and 1.2–1.8 μg/kg/month for those aged 6–11 and ≥12 years, respectively), although 95 % CIs for the children aged <1 year (*n* = 13) were very wide (Fig. 2b).

Throughout the study, the weight-adjusted geometric mean monthly dose was higher in patients receiving dialysis than in patients not on dialysis (Fig. 2c). In patients who were not on dialysis at each time point, the geometric mean DA dose followed a downward trend throughout the study. In patients on peritoneal dialysis at each time point, the geometric mean monthly DA dose was generally higher in the second year of the study than the first, but in hemodialysis patients, values were generally lower in the later part of the study than in the first 6 months.

### Hemoglobin concentrations

In months 1–3, the mean Hb concentration for all patients rose to 11.5 g/dl (from a baseline value of 11.1 g/dl), with little change during the remainder of the study (range from months 4–24 = 11.3–11.4 g/dl). Mean values for each age subgroup are shown in Fig. 2d. The spread of mean Hb concentrations during the study period was similar for patients who were on dialysis at baseline (10.9–11.5 g/dl) and those who were not (11.2–11.7 g/dl).

### Red blood cell transfusions

In all, 48 patients (15.0 %) received at least one RBC transfusion during the study period. Transfusions were more common in children aged <1 year and 1–6 years (3/13 [23.1 %] and 20/83 [24.1 %], respectively) than in the two older age subgroups (12/90 [13.3 %] and 13/133 [9.8 %], respectively). Transfusions by study period and age group are shown in Table 6. RBC transfusions were more common in patients on dialysis at baseline (hemodialysis, 19 [25.7 %]; peritoneal dialysis, 13 [15.5 %]; no dialysis, 16 [10.0 %]).

**Table 6:** Number of patients receiving red blood cell transfusions by age subgroup in each study period. Some patients were transfused more than once during the study. *Values in parentheses* are percentages of the number of patients at the start of the respective period

Time period	Aged <1 year (*n* = 13)	Aged 1–5 years (*n* = 83)	Aged 6–11 years (*n* = 90)	Aged ≥12 years (*n* = 133)
1–6 months	1 (7.7)	10 (12.0)	7 (7.8)	6 (4.5)
7–12 months	1 (10.0)	6 (9.5)	3 (3.8)	3 (2.9)
13–18 months	1 (14.3)	5 (9.6)	5 (7.8)	5 (6.0)
19–24 months	0 (0)	3 (7.0)	2 (4.2)	1 (1.4)
>24 months	0 (0)	0 (0)	0 (0)	0 (0)

The mean pre-transfusion Hb concentration (within the previous 14 days) was 8.9 g/dl, but individual values varied widely (median, 8.4 g/dl; range, 5.7–12.7 g/dl; *n* = 37). The mean pre-transfusion concentration in children aged 6–11 years was lower (8.6 g/dl) than in other children (9.0–9.1 g/dl). Mean pre-transfusion Hb concentration was also lower in dialysis patients than non-dialysis patients (8.8 g/dl and 9.2 g/dl), respectively).

### Other endpoints

During the study, 218 (68.3 %) patients were hospitalized on at least one occasion, with a median length of stay of 16 days (range, 1–180 days). The proportion of children hospitalized tended to be higher in those aged <1 year and 1–6 years (84.6 and 74.7 %, respectively) than in the older age subgroups (60.0 and 68.4 %, respectively). There was little difference in the hospitalization rate by baseline dialysis status. The most common reasons for hospitalization were AEs (113 patients; 51.8 % of all those hospitalized), “normal clinical practice” (75; 34.4 %), and inflammatory conditions (45; 20.6 %).

In all, 229 patients (71.8 %) received supplementary iron during the study (oral, 107; parenteral, 89); the ratio of oral to parenteral iron preparations was similar across all age subgroups. In those on dialysis at baseline, more patients received iron parenterally than orally (*n* = 65 and *n* = 42, respectively), but in those not on dialysis at baseline, the opposite was true (parenteral, *n* = 24; oral, *n* = 65).

## Discussion

To our knowledge, the current observational study is the first to assess safety and tolerability of DA treatment for a period of up to 2 years in anemic children with varying stages of CKD. At baseline, the sample comprised almost equal numbers of children on dialysis and not on dialysis, with the former group containing similar numbers of hemodialysis and peritoneal dialysis patients. For most patients, the study period did not represent the first 2 years of DA therapy, as most had already received DA in the year before study entry. In addition, the range of DA treatment duration during the study varied, with approximately one-half of the enrolled patients withdrawing from the study before the end of the 2-year follow-up.

Approximately 50 % of the children in the study experienced an SAE. This proportion is consistent with a population with deteriorating renal function—approximately two-thirds of the patients required hospitalization on at least one occasion during the study period, and over one-third withdrew after receiving a kidney transplant. It is notable that similar proportions of children have reported SAEs in studies with much shorter follow-up periods. For example, in a 28-week comparison of DA and rHuEpo in children aged 1–18 years, 32/81 (40 %) children on DA reported SAEs, most commonly fever, sepsis, and administration site complications or infections [12], and another 28-week study reported SAEs in 13 (43 %) of 30 children receiving DA [13]. None of the six fatal SAEs reported in the current study was judged by the physician to be causally related to DA treatment.

Six SADRS (i.e., SAEs judged by the treating physician to be associated with exposure to DA), were reported in four children. Although thrombosis and embolism are recognized adverse reactions to DA in adults [10], the SADRs reported in the other three patients are not. In these three patients, limited information was collected on underlying conditions and concurrent medications. The patient who reported temporary blindness suffered from hemolytic uremic syndrome, which was diagnosed before DA treatment started and was considered by the investigator as the cause of the temporary blindness. This patient was on hemodialysis and had ‘chronically elevated blood pressure’ (no blood pressure readings were recorded). In the case of the patient with hemolysis, hemolytic anemia, and thrombocytopenia, DA and other unspecified medications were discontinued, and the events did not recur after reintroduction of DA. The patient with priapism had a history of nephrotic syndrome, which is known to be associated with priapism; however, priapism also occurred in one patient in the rHuEpo comparison study [12], where it was classed as a treatment-related AE. Thus, no new safety signals associated with DA use were identified in this study. The most frequently reported EMIs were hypertension and convulsions (affecting 4.7 and 3.1 % of patients, respectively), both recognized as ADRs in adults with chronic renal failure [10]. Fifteen of the patients with AEs of hypertension or hypertensive crisis had this condition at baseline and were receiving treatment for it.

Although the weight-adjusted dose of DA was generally stable over the 2-year study period, it was slightly but consistently higher in younger than in older children. A possible explanation is that relatively more children aged <6 years were on dialysis at baseline and so required higher doses, which is consistent with the relatively higher requirement for RBC transfusions by the children in this age subgroup compared with children aged 6 years or more. There did not, however, appear to be a difference between age subgroups in the degree of anemia at baseline; the slightly lower mean Hb in children aged <1 year compared with the other children was in line with age-related differences in threshold values for anemia [17].

After rising slightly during the first 3 months following enrollment, mean Hb concentrations remained stable during the remainder of the study. This stability in mean Hb values was seen in all children aged 1 year or older, but in the 13 infants aged <1 year, mean Hb concentrations were noticeably lower in the first 12 months than in the second 12 months. This might simply reflect the natural increase in Hb over 2 years that would be expected in a child aged <1 year at enrollment [18]. Together with the relatively low proportion of patients requiring RBC transfusion (15 %), these findings suggest that DA was effective in managing anemia in these patients. This result is in line with that of a previous study of DA in 39 children aged 11–18 years in which most patients achieved stable Hb concentrations after 6 months, whether they had switched to DA from rHuEpo or were ESA-naive [19], and with the results of two Japanese studies in children aged 1–18 years with CKD who switched from rHuEpo or were ESA-naive [15, 16].

In conclusion, the findings of the current study in a large and heterogeneous population of children and adolescents with CKD, in which 145 patients completed 2 years’ follow-up, add to our understanding of the safety and tolerability profile of DA in this age group and do not suggest any new safety signals in comparison with experience with DA in adults. The stability of DA dose and Hb concentration observed are notable, although the non-interventional design of the study may limit the extent to which these results can be generalized to routine clinical practice.
